# Assessment of Exoskeletons on Nurses’ Quality of Work Life: A Pilot Study at Foch Hospital

**DOI:** 10.3390/nursrep13020068

**Published:** 2023-05-12

**Authors:** Line Farah, Dorota Roll, Amrei Sorais, Alexandre Vallée

**Affiliations:** 1 Innovation Center for Medical Devices, Foch Hospital, 92150 Suresnes, France; 2 Quality of Work Life Department, Foch Hospital, 92150 Suresnes, France; 3 Department of Epidemiology-Data-Biostatistics, Foch Hospital, 92150 Suresnes, France; al.vallee@hopital-foch.com

**Keywords:** nurse, exoskeleton, medical device, quality of work life, assessment, quality of life, musculoskeletal disorders

## Abstract

Background: The prevention of occupational risks is part of the quality of work life and it is a component that improves the physical work environment. The purpose of the present study was to investigate how to maintain posture and to reduce pain and fatigue for nurses, with an exoskeleton adapted to the work at hospital. Methods: The exoskeleton was used between 2022 to 2023 at Foch Hospital, France. Phase 1 consisted of the selection of the exoskeleton, and Phase 2 included the testing of the device by the nurses and a questionnaire to assess it. Results: The “active” ATLAS model from JAPET, ensuring lumbar protection, was selected because it corresponds to all the specification criteria to tackle the nurses’ unmet need. Among the 14 healthcare professionals, 86% were women; the age of the nurses was between 23 years old and 58 years old. The global median satisfaction score of the nurses relative to the use of the exoskeleton was 6/10. The median impact of the exoskeleton on nurses’ fatigue was 7/10. Conclusions: The implementation of the exoskeleton received global positive qualitative feedback from the nurses concerning the improvement of posture and the reduction in fatigue and pain.

## 1. Introduction

The links between work life quality and care quality led National Health Authorities (such as the French Health Authority, HAS) to include work life quality (WLQ) as one of the dimensions to be considered in the certification of hospitals and to systematically integrate it into the quality assessment, as observed in other countries [1,2,3,4,5]. Based on the strong and close link between the well-being of patients and the well-being of caregivers, hospitals have implemented actions to improve the work life quality of their employees, which consequently contributes to improving the quality of patient care [6,7,8,9].

The prevention of occupational risks is part of WLQ, and it is a component that improves the physical work environment [2,10]. Among the occupational risks faced by care providers, musculoskeletal disorders are the leading cause of occupational illnesses and are responsible for significant absenteeism and high compensation costs. A total of 95% of the recognized occupational illnesses of professionals in the care sector are related to work-related musculoskeletal disorders (MSD), with significant financial and economic impact. These MSDs are injuries and disorders that affect the human body’s movement or musculoskeletal system (i.e., muscles, tendons, ligaments, nerves, discs, blood vessels, etc.) [11,12,13]. The work-related factor includes high task repetition, forceful exertions, and repetitive awkward postures, which can be found in the healthcare sector [11]. The bending of healthcare professionals is often associated with rotations. These are high-risk, repetitive, and frequent postures that are held for varying lengths of time for which the caregiver has neither an aid tool nor any adequate protection. Additionally, there are unexpected reactions by patient during care, such as sudden movements, blocks and/or resistance to care. These can surprise the caregiver and generate a reaction, that is, reflex muscle contractions, which are the source of a large number of low back pain cases [11].

The importance of theses musculoskeletal conditions has been recognized as a cause of mortality and morbidity by the United Nations, World Health Organization, and more than 60 countries [14]. The Center for Disease Control and Prevention (CDC) noticed that work-related musculoskeletal disorders (MSD) are a major source of disruption and can lead to a decrease in the performance of workers and companies (decreased productivity and quality) and have a major impact on absenteeism and turnover. In the care sector, this is compounded by the shortage of care workers [15].

From an economic perspective, accidents at work stand for EUR 160 million, and more than 2.3 million workdays are lost in France due to workers’ time off due to injury. Over 60% of the non-attendance at work is related to back pain. In the United States, MSDs represent 29–35% of the occupational injuries related to days away from work [15]. The direct costs were USD 1.5 billion, and the indirect costs were USD 1.1 billion in 2007 in the USA.

Nevertheless, these MSDs are preventable. At Foch Hospital (Suresnes, France), they are the second leading cause of recorded work-related low back injuries. As part of the prevention of this risk, our center has taken preventive measures to limit employees’ exposure to physical work demands, particularly when taking care of patients. Due to the lack of existing solutions, efforts to find protection for caregivers during bed handling led to the possibility of implementing the back-support exoskeletons developed to prevent low back pain [16,17,18]. Exoskeletons are defined as wearable devices designed to help users perform work tasks by applying force to one or multiple joints. There is growing interest in using exoskeletons for workplace ergonomics to reduce physical workload and to lower the risk of musculoskeletal disorders.

Concerning the prevention strategy of MSDs in our centre, the exoskeleton is considered an additional tool that completes the in-house institutional training on “preventing risks related to manual handling by caregivers”, which is regularly provided at Foch Hospital, for instance. This training focuses on a patient-care approach that prioritizes “no lift”, preferring “sliding” over “carrying”, and actively solicits the patient’s participation to the extent of their abilities. The more active the patient’s participation is, the less the caregiver is exposed to the risk of MSDs. On the other hand, the caregiver has different transfer tools to help them in their daily activities such as: (i) The glide sheet for raising a patient, that is, reinstalling them by sliding them towards the head of the bed; (ii) The floor or ceiling rail mobile patient lift for any type of transfer: bed–stretcher or bed–chair for patients who cannot actively participate; (iii) The electric verticalizer for the seated–seated transfer, such as bed–chair, and return for the patients who control the seated position with good lower limb tonicity, etc.

The purpose of the present study was to investigate how to maintain posture and to reduce pain and fatigue for nurses with an exoskeleton adapted to the work at hospital.

The objectives were the following: (1) To choose the suitable exoskeleton that would meet the expectations of the nurses according to the predefined criteria; (i) To maintain posture; (ii) To reduce low back pain and fatigue during their work at hospital; (2) To test the selected exoskeleton and collect the feedback of the nurses on their experience testing the device.

## 2. Materials and Methods

The use of the exoskeleton was performed between January, 2022 and January, 2023 at Foch Hospital, Suresnes, France. The questionnaire was carried out in February, 2023. Two phases were scheduled. Phase 1 consisted of the selection of the exoskeleton, and Phase 2 included the testing of the device by the nurses. Nurses wore the exoskeleton during a total of 36 h to test the device. The study was approved by Foch IRB: IRB00012437 (approval number: 23-03-01), on 13 February 2023. Non-opposed consent was obtained from all participants. We followed the Revised Standards for Quality Improvement Reporting Excellence (SQUIRE 2.0) (Supplementary File S1).

### 2.1. Phase 1: Selection of the Adapted Exoskeleton for Healthcare Professionals

#### 2.1.1. Pilot Group

We conducted a market study of different equipment to address the previously raised problem. Before the testing phase, a pilot group was set up to define the integration methods of the device into the care activity. This pilot group consisted of five multidisciplinary members and included the head of care, care managers, the MSD specialist, and the manager of professional risk prevention. The objectives of this group were to select the device to be tested, to choose the hospital departments, to target the healthcare population, to determine the evaluation criteria, and to ensure the management of the trial.

#### 2.1.2. Specification Requirements to Answer the Unmet Need

The exoskeletons for nurses have specific mechanical features focused on comfort, fitness, and pressure, which differ from the features typically found in industrial exoskeletons. Some of the mechanical features that are specific to healthcare exoskeletons include:

(1) Lightweight design: these exoskeletons are typically designed to be lightweight and comfortable to wear for extended periods of time;
(2) Flexibility and range of motion: they are designed to allow for a wide range of motion, as nurses need to move freely and perform a variety of tasks throughout their shifts;
(3) Pressure distribution: they are designed to distribute pressure evenly across the body, reducing the risk of discomfort or injury.

After assessing different exoskeleton options, two types of models that protect the lumbar region were studied: (i) “Passive” model types provide lumbar support for low “leaning forward” weight carrying, but without horizontal or height lifting assistance, making them logistically better; (ii) “Active” model types have electronic assistance targeting all ranges of motion. In this study, the active model addresses the unmet need of lumbar support.

Therefore, the specification documentation was established to select a device that met the following criteria: (i) Active model; (ii) Compatibility with professional attire; (iii) Adaptation to care environment needs; (iv) Back protection level. The Japet Atlas met these criteria and was selected.

Other types of devices, such as shoulder exoskeletons in particular, have been developed to assist healthcare workers with tasks that involve repetitive overhead movements, such as lifting and transferring patients or reaching for medical supplies on high shelves. These exoskeletons typically consist of a wearable device that is attached to the shoulders and upper back, with motors and sensors that provide assistance to the arms and shoulders during tasks that require overhead movements. However, these devices did not address the unmet need of Foch Hospital.

#### 2.1.3. Presentation of the Selected Low Back Exoskeleton

**Figure 1:**
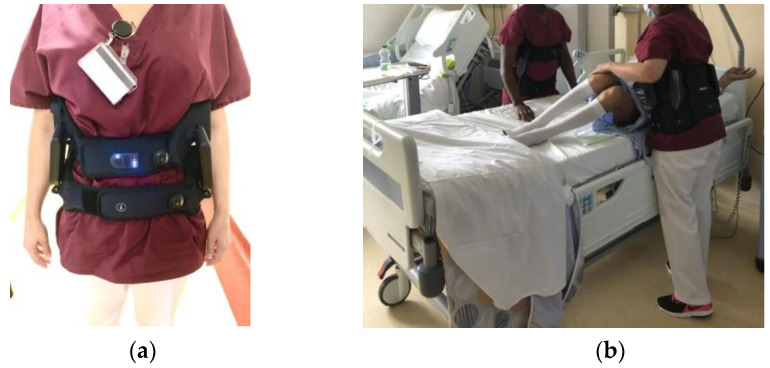
Demonstration of a nurse wearing the JAPET active exoskeleton in anterior view at rest (**a**) and in posterior view during treatment (**b**) at Foch Hospital. This device is controlled by a software system developed by the manufacturer. It is designed to adjust the assistance provided by the motors based on the specific task being performed by the user. The system uses sensors placed throughout the exoskeleton to monitor the user’s movements and provide feedback to the software, which then adjusts the assistance provided by the motors in real time. When the user is walking or standing, the system provides assistance to the legs to help reduce the load on the user’s back muscles. When the user is lifting a heavy object, the system will provide additional assistance to the arms to help support the weight of the object. Overall, the control of the motors for different tasks in the Japet exoskeleton is based on a sophisticated software system that uses sensors and feedback to adjust the assistance provided by the motors in real time, combined with user input through a wireless remote control.

The exoskeleton included a dynamic trunk orthosis (Japet Medical Devices^©^, 59120, Loos, France), which allowed the application of vertical traction forces to diminish the lumbar spine pressure [19]. The Japet Atlas consists of a lumbar belt with rigid shells that take support from the pelvis and prevent moving into risk postures (Figure 1).

The embedded electronics consist of micromotors that operate cylinders, accompanying the trunk movements (flexion, rotation, etc.) by securing all amplitudes. The traction force of the cylinders carries out a stepwise decompression of the intervertebral disks to 40% of the lumbar pressure. The manufacturer’s usage protocol stipulates that the belt must be worn when standing and in regular physical activity. The weight of the belt is 1.850 kg. Different levels of traction force exist, such as 4 kg, 8 kg, 12 kg, and 16 kg. The belt is powered by rechargeable batteries; the charger is connected to a standard electrical outlet, the belt is stored, outside the time of use, in a carrying case. The equipment being expensive, a secure storage place was proposed.

Therefore, the “active” ATLAS model from JAPET, ensuring lumbar protection, seemed to be the device that best tackles the unmet needs of caregivers’ prevention and protection by responding to all the criteria of the specifications.

#### 2.1.4. Selection of the Hospital Departments Testing the Device

The selection of the tests’ departments considers the physical burden of care in relation to the patient’s degree of autonomy. The decrease in the patient’s autonomy increases the harmful efforts and postures of the care giver required to perform certain care tasks. The targeted sectors were the internal medicine department, the intensive care unit, and geriatrics. This required the agreement of professionals, as well as the mutual agreement of the heads of each sector and their respective sector managers, who had a decisive role in motivating their team and remaining vigilant during the progress of the trials.

### 2.2. Phase 2: Test of the Exoskeleton by Nurses at Hospital

The inclusion criteria dictated that the nurses worked at Foch Hospital, were over 18 years old and worked in the following departments: the internal medicine department, the intensive care unit, and geriatrics. The exclusion criterion excluded those for whom carrying loads at work was forbidden.

**Figure 2:**
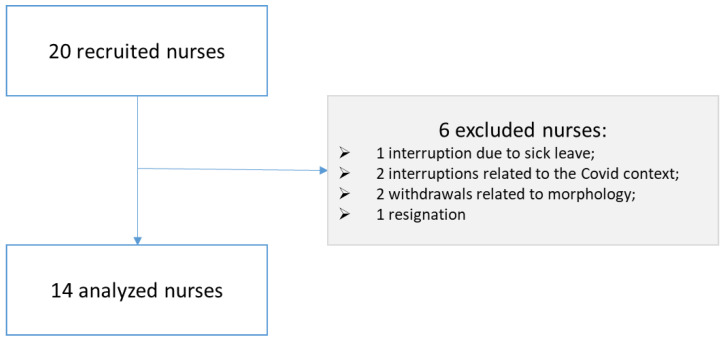
Flowchart of the process of selecting the nurses who tested the exoskeleton.

The process of selecting the nurses is summarized in the flowchart in Figure 2. Out of 20 nurses, 14 were selected to test the device, whereas 6 were excluded (1 due to sick leave; 2 due to to the COVID-19; 2 withdrawals were related to morphology; 1 resignation).

The deployment of the ATLAS belt requires mastering the process of accompanying the user, which is a key element for success. This process involves training in the use of the tool and in the procedures for monitoring and measuring the effects of its use.

We asked the care manager and the MSD specialist of each department to recruit the nurses from the three services selected by the pilot group on a voluntary basis. After the selection, the nurses were included in the study. At the end of each test, the MSD specialist delivered the questionnaire to each nurse. The questionnaire was developed by the pilot group, including five multidisciplinary members (head of nurse care, nurse care managers, the MSD specialist, and the manager of professional risk prevention), who selected the exoskeletons. They designed 20 short items, which were included in the questionnaire and covered 4 areas: (i) Clinical assessment; (ii) Technical and ergonomic assessment; (iii) Nurse satisfaction; (iv) Impact on fatigue.

#### 2.2.1. Data Collection

To assess the device with the targeted caregivers, the data were collected by interviewing nurses on February, 2023, using a 20-item questionnaire covering 4 areas: (i) Clinical assessment; (ii) Technical and ergonomic assessment; (iii) Nurse satisfaction; (iv) Impact on fatigue. The candidates’ eligibility for the study was assessed, and a date was scheduled to train the candidate in the use of the exoskeleton. The questionnaire was given to each participant.

#### 2.2.2. Statistical Analysis

The characteristics of the participants were described according to the level of satisfaction. To assess the usage of this device, the pilot group set a user satisfaction threshold of 7/10, considered adequate for implementing this device to prevent musculoskeletal disorders in nurses. Therefore, we compared the responses of the two groups based on their satisfaction threshold. The continuous variables were described as median and 25th percentile and 75th percentile and compared using the Mann–Whitney test; the categorical variables were described as number and percentage and compared using the Fisher exact test.

## 3. Results

### 3.1. Population Description

Of the 14 nurses, 86% (n = 12) were women. The age of nurses was between 23 years old and 58 years old. The median body mass index was 24.65 (min: 18.6; max: 27.5). The distribution of nurses among the departments was 64% (n = 9) in geriatrics, 29% (n = 4) in the intensive care unit, and 7% (n = 1) in the internal medicine department. A total of 64% of the nurses (n = 9) presented a previous lumbar disease but only 14% (n = 2) had previous pathologies related to musculoskeletal disorders.

### 3.2. Questionnaire Answers

Concerning clinical assessment, 100% (n = 14) of the nurses answered that the exoskeleton maintains their back in a good posture, 93% (n = 13) believed that the device prevents them from taking risk postures, 86% (n = 12) said that the exoskeleton reduces fatigue and lower back pain (Table 1).

Concerning the usability test, the technical and ergonomic aspects were evaluated both at the healthcare professional (HCP) level and the hospital level (Table 1). At the nurse level, all the HCPs believed that the device was easy to adjust, and 93% answered that their medical tools are easily usable with the device. A total of 79% of them highlighted that they are accompanied by the device in their movements during care giving. Even though 72% of the nurses answered that the device was comfortable, 57% of the HCPs highlighted that there was an overheating problem while wearing the device. Moreover, 64% of professionals pointed out that the exoskeleton is not sufficiently discreet. At the hospital organization level, only 64% of the HCPs answered that the exoskeleton is easy to transport, 71% of them believed that the device is light, but only 43% of the HCP considered it sufficiently hygienic.

### 3.3. Nurse Satisfaction and Impact on Fatigue

The median global satisfaction score of the nurses relative to the use of the exoskeleton was 6/10 [min: 3; max: 10], but only 36% of the nurses declared a satisfaction rate above 7/10. The median impact of the exoskeleton on nurses’ fatigue was rated 7/10 [min: 1; max: 9].

Satisfaction was significantly associated with the following items: “device adapted to the work environment” (*p* = 0.037), “all movements are possible with the device” (*p* = 0.005), “movements are performed quickly with the device” (*p* = 0.002), “to be comfortable with other people’s gaze” (*p* = 0.016), and “the device is easy to transport” (*p* = 0.037). We observed a significant correlation tendency between satisfaction and fatigue (*p* = 0.105), comfort (*p* = 0.078), ease of storage (*p* = 0.078), and lightness of device (*p* = 0.078) (Table 1).

## 4. Discussion

This study is a pilot test for the implementation of the exoskeletons to protect nursing professionals against musculoskeletal disorders. The median global satisfaction score of the nurses relative to the use of the exoskeleton was 6/10, with some parameters associated with this satisfaction, such as adapting the exoskeleton to the work environment, all movements being possible and being able to perform them in a rapid manner, and that the device was easy to transport. Moreover, 100% of the nurses declared that the exoskeleton maintained their back in a good posture and was easy to adjust/tighten.

Our study highlighted that the design of the device could ensure lumbar protection from a biomechanical perspective (100% of the nurses responded that the exoskeleton maintained their back in a good posture, and 93% that exoskeleton prevented nurses from taking risk postures), and may reduce fatigue and relieve pre-existing pain (86% of nurses responded that the exoskeleton reduced fatigue and lower back pain), which is consistent with the literature [20,21]. As our study remains a pilot study, this test encourages us to promote the introduction of the exoskeleton in the work life of nurses, leading to a change in work habits. The encouraging and supporting supervision provided to nurses during the adaptation phase reassures them and promotes better adherence to the process. The integration of exoskeletons at Foch Hospital seems to allow correcting errors during dressing, providing posture advice to overcome movement limitations imposed by the belt. This observation remains to be investigated in future studies but seems to be consistent with previously published results in the literature [22,23]. Nevertheless, even if the biomechanical effect is assessed, a psychological effect has been highlighted in some studies, showing a placebo effect in the therapeutic process without specific efficiency but with a positive impact on the patient thanks to psychological mechanisms [24].

Exoskeletons have the potential of supporting and enhancing nurses’ strength for manual handling, thereby reducing physical effort and the risk of musculoskeletal injury. While exoskeletons designed for manual workers handling heavy loads are already available in the market, most of them are geared towards industrial workers, who have different types of tasks and are predominantly men. Nurses, on the other hand, have unique requirements, as they work in hospitals and need to adhere to cleanliness and safety guidelines, which apply to exoskeletons as well. Additionally, the exoskeletons used by nurses must be safe for both the user and the patient, and they should be designed to follow proper handling guidelines. Patient handling tasks can pose a high risk of musculoskeletal injuries due to various factors, such as high force (overexertion), transfer distances, confined spaces, variable patient behavior, awkward postures (stooping, bending, and reaching), and repeated activities (lifting, transferring, and repositioning) [25]. While nurses are most prone to lower back injuries [26], they also commonly suffer from injuries to their neck, shoulders, wrists, and knees [13,27,28]. A survey [29] of 1163 nurses working in the United States revealed that 47% of them had experienced back injuries within the past year, indicating the prevalence of musculoskeletal injuries in this profession.

When designing an exoskeleton for nurses, it is essential to use anthropometry to consider their size, particularly when compared to men workers for whom most modern exoskeletons are designed [16,30]. Ideally, the exoskeleton should be adjustable to fit each user’s size, but this would result in a heavier and more complex design, making it impractical for daily use by nurses. A practical solution would be to design exoskeletons for most nurses, identifying the range of their body sizes. This approach would reduce the weight of the exoskeleton and make it more practical for everyday use in hospital and care home environments [31]. It is worth noting that nursing jobs are predominantly occupied by women. Hence, an exoskeleton design should account for the unique physical requirements of women nurses to ensure that they are adequately supported and protected against musculoskeletal injuries [32].

For an exoskeleton to be widely adopted and assist nurses throughout their workday, comfort is a crucial factor (71% of nurses in our pilot study declared that the device was comfortable). However, achieving comfort remains challenging when an exoskeleton must redistribute pressure to the user’s body [33,34]. Studies have shown that reducing or redistributing pressure on the human body increases comfort and device acceptance [35,36]. A significant association was observed between satisfaction and perceived comfort of the device (*p* = 0.078); thus, to prevent discomfort and possible injuries, the pressure points and contact between the user and the exoskeleton must be well-considered [18].

We observed in our study that 100% of the nurses declared that the exoskeleton was easy to adjust/tighten, and 79% that the exoskeleton was easy to install/disinstall. Thus, it is essential that exoskeletons should be adjustable to fit a wide range body sizes for increased use adherence. Custom-made exoskeletons for each user would be too expensive, but the device should have adjustable attachment sizes to match the user’s size. Research has shown that exoskeletons are more efficient when they fit well to the user’s body [37], which requires careful consideration of human body proportions and measurements.

However, few exoskeletons designed for heavy lifting are adjustable to the average female body [16], as most are designed for men who are manual workers. Women’s body shape and dimensions differ significantly from men’s, and they have different friction points and sensitivity to consider for dynamic movements. Additionally, nurses are rarely involved in exoskeleton design, which could limit the usability and acceptance of exoskeletons in healthcare.

To provide support to nurses throughout their working hours, the exoskeleton should be designed to be comfortable and durable. This notion remains important for the implementation of such devices, as we observed a significant association between satisfaction and declaration of comfort (*p* = 0.078). While passive exoskeletons can be worn for extended periods, some may apply excessive pressure on the user and cause discomfort. On the other hand, powered exoskeletons often require a battery and may not be suitable for prolonged use. Therefore, it is crucial to strike a balance between weight, comfort, and functionality to ensure the exoskeleton can be used effectively by nurses.

To improve ease of use and mobility, the exoskeleton must be lightweight and portable. Heavy exoskeletons can cause unnecessary stress on the user’s body, making them less likely to be used frequently. Additionally, portable exoskeletons would enable nurses to provide care to patients in different areas of the hospital without being constrained by the device’s mobility.

Comfort is a crucial factor in the design of exoskeletons, as it directly affects the user’s willingness to wear it again. However, most exoskeletons are currently perceived as uncomfortable, which hinders their widespread adoption [36]. In addition, some exoskeletons are deemed too bulky to be used effectively in hospitals during close contact with patients [38]. To maximize comfort, an exoskeleton should have an adjustable size and joints that can accurately rotate around the same axis as the user’s joints.

A limitation of the exoskeleton in this study is that the deployment of exoskeletons requires commitment and regular supervision of individual users in the follow-up. This support is time-consuming and requires great availability, especially for night teams. Another limitation is related to the requirement that the user’s mobility relies on the use of the lower limbs. As a result, pathologies affecting the legs’ motor apparatus can be blocking the introduction of the device in the activity of the nurses.

### 4.1. Limitations Section

One limitation of the present study is related to the small sample size of nurses. Therefore, further studies with a higher number of nurses and with a multicentre perspective could help to confirm these preliminary results on a wider population; this is needed to verify the reproducibility of the results.

The age range of the subjects in the present study is relatively broad. The age factor can have a significant impact on human function, which may in turn affect the evaluation of exoskeletons by nurses. Some articles have highlighted that younger individuals may be more receptive to the use of exoskeletons compared to older individuals due to differences in physical abilities and preferences [39].

According to Cha et al., the use of most exoskeletons results in challenges in terms of microbial contamination, compatibility with medical equipment, and mobility in confined spaces. Therefore, exoskeletons may not be suitable for certain tasks in sterile environments, such as operating rooms. Therefore, another limitation is related to the fact that we could not lead explanatory studies in sterile environments and we could not use them for nurses in operating rooms, for instance [40].

Although quantitative outcomes were collected from participants, collecting participants’ comments and feedback would constitute an interesting qualitative analysis for further study. The present study did not assess the impact of the long-term use of exoskeletons on nurses, and additional long-term studies are needed to validate the reproducibility of the results.

The majority of the overall expenses are made up of indirect costs, since back pain accounts for 30% of work interruptions lasting more than 6 months and 20% of occupational accidents resulting in work stoppages lasting more than two months, across all sectors [41]. Therefore, an additional long-term health economic evaluation on a higher number of nurses could improve our understanding on the overall impact of these exoskeletons on the nurses’ quality of work life. Other areas of interest regarding potential uses of exoskeletons are assessed for technical jobs at hospitals [19].

### 4.2. Recommendations

This experiment leads us to some recommendations related to the use and deployment of exoskeletons for healthcare professionals:

1. Target a specific population of caregivers:

Select the healthcare professionals for whom the exoskeleton would be appropriate to meet a need, while they can support the requirement of the device in terms of weight, height, mobility;

2. Rethink the design of the device and define relevant specifications to adapt it to the healthcare environment:

Use a material that allows HCPs to feel less heat and modify the design to help the nurses move quickly and easily;

3. Prevent the development of lower limb conditions such as ankle arthrodesis and knee problems:

Integrate the exoskeleton in the prevention strategy of MSDs of your centre as an additional tool that addresses unmet needs not already addressed by the existing tools.

## 5. Conclusions

The implementation of exoskeletons received positive qualitative feedback from the nurses concerning the improvement of posture and the reduction in fatigue and pain, despite some points of improvement being highlighted by the users during the test phase. Additional qualitative (with semi-structured interviews) and multicentre studies on a larger number of nurses are still needed to confirm these results and to understand the reason of the nurses’ satisfaction. A health economic analysis on a larger number of nurses is also needed to assess the impact of these exoskeletons on the nurses’ quality of work life.

## Figures and Tables

**Table 1:** Questionnaire answers by the nurses who tested the exoskeleton.

	Overall Population (N = 14)		Satisfaction > 7 n = 5		Satisfaction < 7 n = 9		
	N/Median	%/IQR	N/Median	%/IQR	N/Median	%/IQR	*p* Value
**Clinical assessment**							
Age (years)	32	[25–46]	27	[25–48]	33	[26–49]	0.751
Sexe (female)	12	85.71%	4	80.00%	8	88.89%	0.649
BMI (kg/m^2^)	25	[24–26]	25	[23–27]	25	[23–26]	0.821
Fatigue	7	[5–8]	8	[7–9]	6	[5–7]	0.105
Does the exoskeleton maintain the back in a good posture? (yes)	14	100.00%	5	100.00%	9	100.00%	1.000
Does the exoskeleton prevent me from taking risk postures? (yes)	13	92.86%	5	100.00%	8	88.89%	0.439
Does the exoskeleton reduce fatigue and lower back pain? (yes)	12	85.71%	5	100.00%	7	77.78%	0.255
**Technical and ergonomic assessment**							
***At nurse level***							
Is the device adapted to my work environment?	9	64.29%	5	100.00%	4	44.44%	0.037
Are all movements possible with the device?	7	50.00%	5	100.00%	2	22.22%	0.005
Do I perform movements quickly with the device?	4	28.57%	4	80.00%	0	0.00%	0.002
Am I accompanied by the device in my movements?	11	78.57%	4	80.00%	7	77.78%	0.923
Are my tools easily usable with the device?	13	92.86%	5	100.00%	8	88.89%	0.439
Is the device comfortable?	10	71.43%	5	100.00%	5	55.56%	0.078
Is heat a problem for you?	8	57.14%	2	40.00%	6	66.67%	0.334
Is the device sufficiently discreet?	5	35.71%	3	60.00%	2	22.22%	0.158
Am I comfortable with other people’s gaze?	8	57.14%	5	100.00%	3	33.33%	0.016
Is the device easy to adjust/tighten?	14	100.00%	5	100.00%	9	100.00%	1.000
Is the device easy to install/uninstall?	11	78.57%	4	80.00%	7	77.78%	0.923
***At the hospital organization level***							
Is the device hygienic?	6	42.86%	3	60.00%	3	33.33%	0.334
Is the device easy to store?	10	71.43%	5	100.00%	5	55.56%	0.078
Is the device easy to transport?	9	64.29%	5	100.00%	4	44.44%	0.037
Is the device light?	10	71.43%	5	100.00%	5	55.56%	0.078

IQR: interquartile range (25th percentile and 75th percentile).

## Data Availability

Data collected during this study are available by a request to the authors.

## Supplementary Materials

The following supporting information can be downloaded at: https://www.mdpi.com/article/10.3390/nursrep13020068/s1.
