# Supplementary material for: Assessment of Exoskeletons on Nurses’ Quality of Work Life: A Pilot Study at Foch Hospital
Source: Nurs Rep. 2023 May 12;13(2):780–91. doi: 10.3390/nursrep13020068 (PMC10204513; doi:10.3390/nursrep13020068)
Supplement: Supplementary file 1 [file nursrep-13-00068-s001.zip › nursrep-2290530-supplementary.pdf]

Supplementary File S1: Table: Revised Standards for Quality Improvement Reporting Excellence (SQUIRE 2.0)

Text Section and Item Name	Section or Item Description	Page(s) of the manuscript
**Title and Abstract**		
**1. Title**	Indicate that the manuscript concerns an initiative to improve healthcare (broadly defined to include the quality, safety, effectiveness, patient- centeredness, timeliness, cost, efficiency, and equity of healthcare)	**1**
**2. Abstract**	a. Provide adequate information to aid in searching and indexing<br>b. Summarize all key information from various sections of the text using the abstract format of the intended publication or a structured summary such as: background, local problem, methods, interventions, results, conclusions	**1**
**Introduction**	*Why did you start?*	
**3. Problem Description**	Nature and significance of the local problem	**2; 3**
**4. Available knowledge**	Summary of what is currently known about the problem, including relevant previous studies	**2; 3**
**5. Rationale**	Informal or formal frameworks, models, concepts, and/or theories used to explain the problem, any reasons or assumptions that were used to develop the intervention(s), and reasons why the intervention(s) was expected to work	**2; 3**
**6. Specific aims**	Purpose of the project and of this report	**3**
**Methods**	*What did you do?*	
**7. Context**	Contextual elements considered important at the outset of introducing the intervention(s)	**3**
**8. Intervention(s)**	a. Description of the intervention(s) in sufficient detail that others could reproduce it<br>b. Specifics of the team involved in the work	**4; 5**
**9. Study of the Intervention(s)**	a. Approach chosen for assessing the impact of the intervention(s)<br>b. Approach used to establish whether the observed outcomes were due to the intervention(s)	**5-7**

**10. Measures**	a. Measures chosen for studying processes and outcomes of the intervention(s), including rationale for choosing them, their operational definitions, and their validity and reliability<br>b. Description of the approach to the ongoing assessment of contextual elements that contributed to the success, failure, efficiency, and cost<br>c. Methods employed for assessing completeness and accuracy of data	**6; 7**
**11. Analysis**	a. Qualitative and quantitative methods used to draw inferences from the data<br>b. Methods for understanding variation within the data, including the effects of time as a variable	**6; 7**
**12. Ethical Considerations**	Ethical aspects of implementing and studying the intervention(s) and how they were addressed, including, but not limited to, formal ethics review and potential conflict(s) of interest	**3**
**Results**	*What did you find?*	
**13. Results**	a. Initial steps of the intervention(s) and their evolution over time (*e.g.*, time-line diagram, flow chart, or table), including modifications made to the intervention during the project<br>b. Details of the process measures and outcome<br>c. Contextual elements that interacted with the intervention(s)<br>d. Observed associations between outcomes, interventions, and relevant contextual elements<br>e. Unintended consequences such as unexpected benefits, problems, failures, or costs associated with the intervention(s).<br>f. Details about missing data	**7; 8; 9**
**Discussion**	*What does it mean?*	
**14. Summary**	a. Key findings, including relevance to the rationale and specific aims<br>b. Particular strengths of the project	**9**

**15. Interpretation**	a. Nature of the association between the intervention(s) and the outcomes<br>b. Comparison of results with findings from other publications<br>c. Impact of the project on people and systems<br>d. Reasons for any differences between observed and anticipated outcomes, including the influence of context<br>e. Costs and strategic trade-offs, including opportunity costs	**9; 10; 11**
**16. Limitations**	a. Limits to the generalizability of the work<br>b. Factors that might have limited internal validity such as confounding, bias, or imprecision in the design, methods, measurement, or analysis<br>c. Efforts made to minimize and adjust for limitations	**11**
**17. Conclusions**	a. Usefulness of the work<br>b. Sustainability<br>c. Potential for spread to other contexts<br>d. Implications for practice and for further study in the field<br>e. Suggested next steps	**12**
**Other information**		
**18. Funding**	Sources of funding that supported this work. Role, if any, of the funding organization in the design, implementation, interpretation, and reporting	**12**
